# Applications of Flow Cytometry to Characterize Bacterial Physiological Responses

**DOI:** 10.1155/2014/461941

**Published:** 2014-09-09

**Authors:** Verónica Ambriz-Aviña, Jorge A. Contreras-Garduño, Mario Pedraza-Reyes

**Affiliations:** Departamento de Biología, División de Ciencias Naturales y Exactas, Universidad de Guanajuato, Noria Alta S/N, 36050 Guanajuato, GTO, Mexico

## Abstract

Although reports of flow cytometry (FCM) applied to bacterial analysis are increasing, studies of FCM related to human cells still vastly outnumber other reports. However, current advances in FCM combined with a new generation of cellular reporter probes have made this technique suitable for analyzing physiological responses in bacteria. We review how FCM has been applied to characterize distinct physiological conditions in bacteria including responses to antibiotics and other cytotoxic chemicals and physical factors, pathogen-host interactions, cell differentiation during biofilm formation, and the mechanisms governing development pathways such as sporulation. Since FCM is suitable for performing studies at the single-cell level, we describe how this powerful technique has yielded invaluable information about the heterogeneous distribution of differently and even specialized responding cells and how it may help to provide insights about how cell interaction takes place in complex structures, such as those that prevail in bacterial biofilms.

## 1. Introduction

The study of bacterial physiological responses using approaches that assess the overall population response considering it as homogeneous is becoming a thing of the past. Current experimental evidence indicates that an ordinary laboratory culture considered as being composed of isogenic bacteria is actually constituted of heterogeneous subpopulations [1, 2] that respond differentially to changes in their environment [3]. Although an invaluable amount of knowledge has been obtained by studying batch cultures, it should be considered that intracell heterogeneity and cell-to-cell interaction (e.g.,* quorum sensing*) exist in an isogenic bacterial culture. Therefore, analysis focused on subpopulations combined with single-cell analysis techniques will help progress our understanding of the bacterial subpopulation behavior in laboratory cultures.

Current available technologies like flow cytometry (FCM) provide more information regarding the individual events that may rule out the overall population response [4]. As reviewed and discussed by Lidstrom and Konopka [5], a normal distribution in the response across the bacterial cells in a culture does not exist, but there are* on*,* off*, and* intermediate* states that depend on a threshold response mechanism, giving rise to physiologically distinct populations. The extent or probability that a well-differentiated subpopulation response could impact on the overall population behavior or fate of a bacterial culture will depend on the nature of the response itself. For instance, when the subpopulation produces and secretes an inhibitor, a growth factor, or an autoinducer into the medium, it causes a response from the rest of the cells [6]. The origins of such heterogeneity are, according to the literature [7], due to differences in microenvironments [8], created by a large number of bacterial cells* growing in* and* modifying a* culture, giving rise to what is called extrinsic noise, in combination with intrinsic cellular noise, due to the fact that each cell possess a different spatiotemporal distribution of cellular components, that is, the threshold levels of molecules that switch* on/off* gene expression arose at different times in each cell, since gene expression is stochastic [9].

In this context, if we are interested in assessing a bacterial response to certain stimuli, using a fluorescent tracer, and we find no difference between the treated and non-treated samples, it may be due to an absence of response or perhaps to a response in a few percentage of the cells. In a distinct scenario, we could measure the treated culture, if we find in the treated sample half the fluorescence compared to the non-treated sample, we may interpret that all the cells diminished the response by half or perhaps that half the cells do not fluoresce at all or perhaps all the intermediary situations. In order to solve this question, it is necessary to measure the fluorescence emitted by each cell in the culture.

FCM is a useful tool for accomplishing this purpose; this technology was first used in the 1960's [10], and since then a huge increase in its development and applications in different fields can be found in the literature. In 1996, Davey and Kell wrote a comprehensive review about the application of FCM in studying heterogeneous microbial populations [10]; therefore, the present review is primarily focused on the information published after that report, emphasizing the applications of FCM when studying bacterial physiological responses.

## 2. Application of Flow Cytometry

### 2.1. Flow Cytometry: An Overview

**Figure 1:**
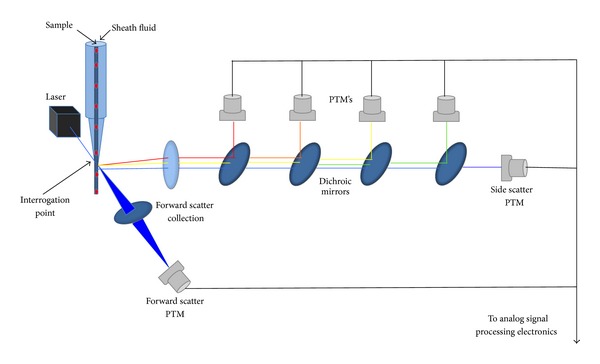
Drawing of the main elements that compose a flow cytometer.

A flow cytometer is an apparatus that makes cells or micrometric particles pass through an interrogation point, where a laser beam impacts them and the light that the particle absorbs, scatters or emits due to its intrinsic or extrinsic physical properties are measured. There are a variety of bibliographic resources [11, 12] that explain at length how a flow cytometer works, but for practical purposes, here we only present a brief and simple description. A flow cytometer is composed of three major parts: fluidics, optics, and electronics systems (Figure 1). The goal of the fluidics system is to make the cell or particle of interest pass through the interrogation point, one by one, in the center of a core stream, within which the diameter of the particle's trajectory is controlled by means of the laminar flow exerted by a surrounding sheath stream so that ideally just one cell passes at a time. The optics system consists of a light source: the most typical one is a 488 nm, (blue) Argon ion laser; this beam is focused by means of lens to impact on the cell in the interrogation point. The particle under study scatters light to all the angles, and the light scattered at acute angles, called forward scatter, is (in general) indicative of a particle's size. The light scattered at wide angles (90°), which is called side scatter, is proportional to the particles' roughness and complexity. Forward scattered light is detected in front of the incident laser beam trajectory by a photodiode or by a photomultiplier tube (PMT), and side scattered light and fluorescence are collected, collimated, and directed 90° through a pathway between a series of dichroic mirrors or beam splitters that permit the pass of certain, unwanted wavelength light and reflect the wanted light to a band pass filter (red, orange, yellow, and green light) and finally to a PMT (Figure 1). In the electronics system, the PMT collects photons and expels electrons, amplifying the signal because a PMT may produce a few hundred electrons for each photon. The photo detectors then produce brief current pulses that are amplified, converted to voltage, and finally converted to numbers by the analog signal processing electronics. There are several ways to represent all the hundreds of events generated per second, and ultimately, although the user knows that he puts in cells in a flow cytometer, after the analysis he obtains a series of numbers and plots to interpret [11]. Here is where a subsequent fluorescence activated cell sorting (FACS) and/or complementary (e.g., microscopy) analysis would be useful.

As noted above, FCM provides useful information regarding the intrinsic characteristics of a cell or particle; thus, the amount of light scattered at low angles or forward scattering (FS) keeps in most cases a direct relationship with the size of the particle, whereas the complexity, granularity, and protein content can be generally estimated on basis of the light scattered at high angles, a property referred to as side scattering (SS) [11]. Although light scattering is a function of intrinsic characteristics of cells it is difficult to distinguish between different bacterial species employing only this property, therefore, to accomplish this task, cells must be marked with fluorescent dyes [13]. However, light scattering has been successfully used to determine distinct biological properties in bacteria, including, poli(*β*-hydroxybutyrate) accumulation [14, 15] as well as cell filamentation and death [16, 17]. Moreover, changes in light scattering has been successfully used for monitoring and sorting the best clones in an* E. coli* culture capable of producing interferon or growth hormone [18]. The capacity of conventional flow cytometers to measure intrinsic and/or extrinsic fluorescence in cells has been used as a property to assess cell viability, protein identity, and enzymatic activity [10, 11, 19]. Discussions regarding the specific properties of fluorescent probes have been published elsewhere [10–12]. Moreover, a complete list of fluorescent dyes can be found in the catalog of Molecular Probes [20]. Readers can find the most appropriate dye for specific FCM needs using these resources, including the target, the type of cell under study, and the excitation/emission wavelengths of the fluorescent compound. One step that may be problematic for FCM analysis during staining is that permeabilization and fixation may affect the viability of the cells; specific examples of how these problems can be overcome have been previously published [16]. Gene expression analysis in bacteria has been assessed by FCM employing fluorescent proteins including the* Aequorea* green fluorescent protein (GFP) [21]. Special forms of GFP have been specifically tailored for its application in FCM [22, 23].

The small size of the bacteria could pose a limitation for FCM analysis specifically for the difficulty of distinguishing between small cells and cellular debris. To overcome this, the literature recommends using both forward scatter and fluorescence as dual trigger signals [10, 11]. Moreover, nucleic acid staining for distinguishing between cells and abiotic particles and the employment of polymer beads for standardization have been found useful in overcoming this limitation [16, 17, 24]. Finally, cell aggrupation, including the formation of bacterial chains or clusters of cells, may be problematic during FCM analysis since this technique measures events and cannot distinguish between a single cell and groups of cells passing through the interrogation point. Therefore, it is necessary to disaggregate and homogenize the sample before analysis; mild sonication has been applied to accomplish this purpose [25–27].

**Figure 2:**
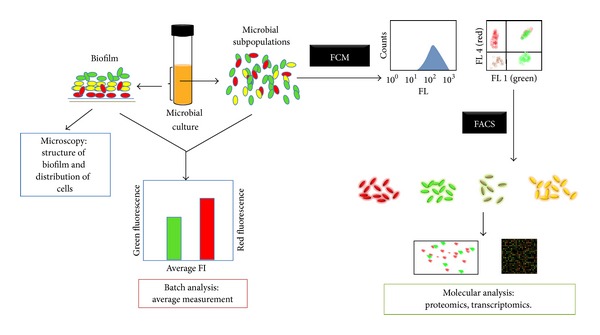
*Left*. Structure and cell distribution in biofilms can be studied by microscopy.* Bottom left*. Analysis of a bacterial culture sample allows obtaining average measurements of a physiological response.* Right*. FCM allows analyzing subpopulations of cultured bacterial cells with different physiological states; FACS permits separating those subpopulations that can be independently characterized by high throughput molecular techniques.

Fluorescence activated cell sorting (FACS) is an invaluable tool for separating the subpopulation(s) of interest that possess certain measurable characteristics. A cell sorter is basically a flow cytometer that has the option of separating cells. When the cell sorter detects a particle with the characteristics chosen by the operator, a charge is given to the droplet where that particle is, and as the droplets are passing through an electrostatic field formed between two charged plates, charged droplets are deflected appropriately, whereas uncharged droplets continue on their original course to the waste tank. With this methodology, around 40 000 cells can be separated per second [28]. As shown in Figure 2, what makes this technique relevant is the possibility of separating subpopulations for subsequent physiological and/or molecular analysis [29, 30]; moreover, cells can be recovered alive for growth and physiological analysis purposes [31]. However, if the cell population of interest exists in a very low abundance, it may be very difficult in principle to set the correct gate for sorting it. Secondly, it may take a long time to obtain a useful quantity of cells for downstream analysis; for example, at least 1 000 to 10 000 cells may be needed for obtaining detectable PCR products [30], and if the population of interest constitutes 0.1% of the total sample, it would take us approximately 4 minutes to obtain 10 000 cells at a sorting rate of 40 000 cells per second, and it would take us up to 41 minutes to obtain the sufficient number of cells if the abundance of such population is as low as 0.01%. Furthermore, to perform proteomics, up to 5 × 10^6^ to 10^9^ bacterial cells may be required, and it took 3 days and 3 weeks, respectively, to collect that number of cells of interest [29, 32, 33]. Both the time and gating problems have proven to be diminished by repeated sort cycles, enriching first the population of interest using a wide gate and after that using a narrower gate to purify it [30]. Otherwise, if we are interested in certain subpopulation that possess a transient physiological state for further analysis, the lapse of time required for separate those cells could be too long that the transient phenotype could be missed before reaching the number of cells needed.

Although FCM emerged from 1960's as a tool for analyzing blood cells, advances in flow, optics, electronics, and probing techniques have done this approach suitable for study of a great number of purposes in different biological systems [12]. Quixabeira et al. [34] made an analysis of published literature regarding FCM applied to genetic studies, from 1991 to 2007, and they reported that more than a half of these studies are human related, the majority are related to diseases, and just a small proportion has been devoted to studying bacterial responses (viruses/bacteria, 8.4%). The main factor that has limited the use of FCM in prokaryote field is the small size of bacteria which makes them difficult for detection [4, 34, 35]. However, current improvements have allowed applying this technique to characterize the physiological events underlying the bacterial responses as described below.

### 2.2. Flow Cytometry Analysis of Bacterial Response to Antibiotic Agents

Because an increasing number of resistant strains are emerging [36, 37], huge efforts are dedicated to new antibacterial drug research [38, 39] using automated methods to assess the number of candidates required, besides the necessity to find in terms of hours rather than days the appropriate drug for treating bacterial infections in clinical practice [40, 41].

When studying response to antibiotic agents, susceptibility is traditionally assessed in bulk, by the growth capability of the bacteria. It is possible to study viability as a parameter of antibiotic susceptibility at single-cell level, measuring criteria such as impermeability of membrane to dyes, maintenance of membrane potential, and the presence of metabolic activity measured by the production of a fluorescent metabolite from a nonfluorescent precursor [12].

Walberg et al. [24] employed FCM to assess the susceptibility of growing* E. coli* cells to mecillinam and ampicillin by using DNA staining with a combination of mithramycin and ethidium bromide (first reported by Steen et al., 1994 [42]). Prior to staining, the cells had to be permeabilized with ice-cold ethanol treatment. Fluorescence was recorded to measure DNA content, forward scatter recorded as cell size indicative, and side scatter as proportional to dry weight or protein content. These three parameters were augmenting in cells exposed to antibiotic since 30 minutes (and doubled at 60 min) incubated with minimal inhibitory concentration (MIC) of mecillinam and ampicillin. Thus showing that DNA and protein synthesis continued but cells were unable to complete division, since cell number remains constant. Such results were in agreement with the known mode of action of penicillin, which interferes with cell wall synthesis. Walberg's results indicate that, under the conditions tested, drug responses can be detected by light scatter alone, without staining the cells, but, as Walberg points out, in clinical samples, fluorescence measuring is important in order to distinguish between cells and other particles like debris. In an ongoing study [16], the same group demonstrated the applicability of flow cytometry measurement of DNA content and light scattering to assesses the susceptibility of antibiotic drugs, with different modes of action, including, ceftazidime a beta lactam that works as a cell wall antagonizer, ciprofloxacin, a quinolone that targets DNA gyrase, and gentamicin, an aminoglycoside that irreversibly binds to ribosomes. Their results, supported by microscopic observations, were consistent with the action mode of the tested drugs. They reported for these drugs that the cell size/dry weight ratio and DNA content augment as a function of drug concentration and exposure time. The same was true in fluorescence versus forward scatter plot with the existence of populations of filamenting cells, disintegrating filaments, and debris after treatment with the drugs. In a subsequent study, Walberg et al. [17] reported the applicability of the previously exploited technique in studying a heterogeneous drug response, to a mixed culture of two clinically important bacteria species:* E. coli* and* Klebsiella pneumoniae*. These microorganisms are associated with polymicrobial urinary tract infection and both have different susceptibility to the drug tested, ampicillin [17]. This study was found to be valuable in detecting polymicrobial infections; by measuring cell number, light scattering, and DNA content-associated fluorescence, the findings show that it was possible to detect susceptible and resistant cells within an hour of incubation with ampicillin in the same sample.

In another study, Dessus-Babus et al. developed a flow cytometric method to calculate the minimal inhibitory concentration (MIC) to distinct antibiotics in clinical isolates of* Chlamydia trachomatis* and a reference strain [43]. They infected McCoy cells and later treated them with increasing doses of doxycycline, ofloxacin, and erythromycin; after incubation with the antibiotic, they immunostained McCoy cells with a fluorescein isothiocyanate- (FITC-) conjugated antibody to detect* Chlamydia* inclusions. Dessus-Babus and colleagues were able to quantify inclusion forming colonies (IFC)/mL by microscopy and by FCM; the latter method was not as sensitive as microscopy, but it did have the advantages of being specific and reproducible. Although infected McCoy cells were quantified instead of bacterial cells, this study demonstrated that flow cytometry is useful because the interpretation of results did not depend entirely on a skilled and experienced observer. Moreover, since FCM is an automated method, it is time-saving and results can be statistically improved.

Another application of flow cytometry to determine MIC of antibiotic drugs was reported by Assunção et al. in 2007. These authors succeeded in determining the MIC of nine different antibiotics in* Mycoplasma hyopneumoniae*, quantifying the total number of cells by means of staining DNA with SYBR Green. The results obtained with this approach were comparable with those obtained by the classical broth dilution method, which is dependent on the observation of a color change or increasing turbidity in the growth medium. However, the FCM approach was faster (12 h) than the broth dilution method (48 h), and furthermore, it was concluded that at 48 h FCM was more sensitive for tylosin and at 72 h for oxytetracycline and streptomycin MIC determination [44].

Recently, Soejima et al. reported for the first time an FCM based methodology to distinguish between live-injured and dead* Listeria monocytogenes* cells after treatment with antibiotics [45]. Furthermore, these authors were able to identify both states of bacterial cells in clinical blood samples, revealing the potential value of this approach to the opportune evaluation of bacteremia and the assessment of drug treatment in real time. This methodology employed photoactivated ethidium monoazide (EMA) that cleaves DNA of injured, not living cells, in combination with SYTO9 that enters live and dead cells and PI that penetrates dead cells that had lost membrane integrity [45].

Besides susceptibility to antibiotic assessment, relevant information can be obtained by FCM analysis in order to establish antibacterial drug action mechanisms [46–51]. Such information cannot be obtained by traditional culture-based techniques [52]. The use of fluorescent probes to detect specific cell changes [12], including, permeabilization and changes, in membrane potential [53–55], DNA content [16, 17, 24], and metabolic activity [53, 55], are useful parameters to assess viability and thus antibiotic susceptibility. Thus, Suller and Lloyd [52] performed a study to evaluate the effects of ceftazidime on* Pseudomonas aeruginosa*, ampicillin on* E. coli*, and vancomycin on* Staphylococcus aureus*, using the fluorescent probes bis(1,3-dibutylbarbituric acid), trimethine oxonol (DiBAC_4_(3)), and SYTOX Green to measure membrane potential, the redox dye cyano-2,3-ditolyl tetrazolium chloride (CTC) to measure actively respiring bacteria, and the Baclite viability kit (Molecular Probes, Life Technologies, Grand Island, NY) to test viability. Results showed that the use of these fluorophores is effective to assess the antibacterial activities of the drugs tested although different responses between dyes were apparent. Thus, CTC was more efficient in detecting different subpopulations than DiBAC_4_(3) and SYTOX Green when fluorescence values were plotted as a function of forward scatter; moreover, when the dyes (SYTO9 and propidium iodide) of the Baclite viability kit were used, no populations of differently responding cells were detected. Additionally, the use of these dyes has the advantage that no pretreatments of cells were required. Other studies have succeeded in employing fluorescent probes to measure the efficiency of new antibacterial drugs [12, 56, 57]. For instance, Ghosh et al. [57] demonstrated by FCM the antibacterial activity of two bioactive compounds isolated from seeds of* Alpinia nigra* against seven pathogenic bacteria. Ghosh and colleagues found by measuring of the extent of propidium iodide DNA staining of treated cells that both compounds caused significant increase in fluorescence intensity compared to the controls, which indicates membrane damage. The interpretation of this finding was supported by field emission scanning electronic microscopy; they observed membrane disintegration and significant damage to the cell wall [57].

Since multiparametric flow cytometric analysis gives precise, reproducible, and accurate information regarding cell function at a single-cell level, the validity of growth based methods to assess cell viability has been questioned [58]. However, it has also been questioned whether cytometric measurements correlate with cell viability in the culture [12]; thus, it is reasonable to emphasize that there is no a universal formula that can be applied to every bacteria and every drug, but it is clear that FCM is a very sensitive, accurate, and time-saving method for assessing bacterial responses to antibiotics [16, 50]. Inconsistencies between cytometric analysis and growth based approaches can be explained by the existence of metabolically active but noncultivable cells (VNBC) in a cell culture [58, 59]. In fact, FCM has been successfully applied to detect VBNC cells from a variety of sanitary and clinically important sources, when the isolation and growth of viable cells are not possible [60, 61]. Cell sorting (by FACS) and further analysis of cells allows us to do further tests in order to corroborate the cytometric results with traditional methods, such as growth in culture and high-resolution single-cell microscopic analysis [58, 62].

As noted above, GFP reporter fusions are very useful for studying gene expression, and Sánchez-Romero and Casadesús [63] quantified GFP fluorescence by flow cytometry, in a liquid isogenic culture of* Salmonella enterica* expressing a* ompC:gfp* reporter gene fusion. In this study, heterogeneity was observed in the expression levels of the outer membrane porin coding gene; moreover, after sorting populations with high and low expression levels and assessing the susceptibility of such cell populations to kanamycin, it was found that low levels of* ompC* expression correlate with high kanamycin resistance. They proposed that the noisy expression of* ompC* is a mechanism contributing to the adaptive resistance to lethal concentrations of kanamycin [63]. In another study, Cui et al. used a GFP fusion as reporter of* graF* expression; the researchers sorted* S. aureus* cells with different levels of fluorescence by FACS, further susceptibility analysis to glycopeptide antibiotics, and morphology studies of* graF* overexpressing cells were performed. They found that upregulated activity of* graF* promoter is consistent with reduced susceptibility to glycopeptides due to an increased cell wall thickness [64]. Another approach to detect resistant bacteria to antibiotics is using fluorescent drug analogs called reporter enzyme fluorescence [65, 66] or using drug fluorescent analogous with FRET pairs (fluorescence resonance energy transfer), that upon enzyme cleavage, the quenching molecule is eliminated and the fluorophore then emits light [67, 68]. An example of this approach was reported by Shao et al. who designed optical probes for detecting *β*-lactamase activity and covalent fluorescent labeling of antibiotic-resistant bacteria [69]. Therefore, an increasing number of future applications in research and clinical fields using the above-mentioned techniques may be anticipated, which can be substantially potentiated by employing personalized probes or dyes for monitoring particular targets and cellular functions.

### 2.3. Measurement of Bacterial Responses to Other Chemical and Physical Stresses

Together with the search for chemical agents that are useful to kill bacteria, other strategies have also considered the idea of causing cell stress and stopping bacterial growth. One of such approaches employs photodynamic inactivation (PDI), which consists of the use of a nontoxic photosensitizer that is activated by harmless visible light. This then produces reactive oxygen species that causes fatal damage to target bacteria [70]. This approach has been tested* in vitro* [71] and* in vivo* [72]. Since PDI causes nonspecific injuries to the pathogens, it is very unlikely that bacteria can acquire resistance. One work related to ROS production by photoactivation of hypocrellin B (a component of a traditional Chinese herb* H. bambuase*) was reported by Jiang et al. [73]. In this report, clinically isolated* S. aureus* cells were incubated with hypocrellin B and after photodynamic treatment they used DCFH-DA (dichlorodihydrofluorescein diacetate) to measure by FCM the production of ROS [74]. ROS levels increased substantially in cells treated with PDI. The treatment caused reduction of viability as demonstrated by CFU counts and cellular damage, including membrane damage and cytoplasm leakage that was corroborated by confocal laser scanning microscopy [73].

FCM has also been applied in assessing the effectiveness of the treatments that food products for human consumption receive to eliminate and prevent pathogen dissemination and spoiling bacteria, including* Listeria monocytogenes*. This bacterium possesses a remarkable adaptability to stress conditions during food processing such as acidic pH, high salt concentration, and extreme temperatures [75]. It has been shown that, in order to cope with these stresses, this microorganism activates the expression of genes from the general stress sigma B (*σ* ^B^) regulon. To clarify the role of *σ* ^B^ during adaptation to low temperature, Utratna et al. analyzed the expression of a reporter gene fusion between the promoter of the *σ* ^B^-dependent* Imo2230* gene and GFP at normal and low temperatures. The FCM analysis revealed a heterogeneous activation of the *σ* ^B^-dependent GFP fusion expression occurring since the early exponential phase, although the maximal expression occurred when cells entered into the stationary phase, of growth; however, such a result was found to occur at both temperatures tested. Therefore, they concluded that *σ* ^B^ does not play a pivotal role in adaptation to cold temperatures [76].

Another bacterium receiving a lot of attention in regard to stress response characterization, being a causal agent of food borne diseases, is* Bacillus cereus* [77]. Flow cytometry among other techniques has been useful in studying* B. cereus* responses to several chemical and physical stresses, such as low pH, which are encountered by the bacterium in several situations including the treatment that alimentary products are subjected to [78], in the host's gastrointestinal system [77, 79], and to other sporistatic/sporicidal physicochemical treatments [80, 81].

FCM has also been applied to investigate the way in which bacteria respond and adapt to stressful conditions that they face during industrial and remediation bioprocesses [82].* Streptococcus macedonicus*,* Bacillus licheniformis*, and* Lactobacillus rhamnosus* are examples of bacteria employed in industrial fermentations. Papadimitriou et al. [55] reported the use of FCM to assess* in situ* the physiological status of* S. macedonicus* in response to acid stress. This microorganism that is a member of the lactic acid bacteria group is widely used in food industry. In this report, Papadimitriou and colleagues employed FCM to analyze characteristics such as membrane potential with DiBAC_4_(3), membrane integrity with propidium iodide, and enzymatic activity as well as membrane integrity with cFDA, carboxyfluorescein diacetate. Finally, they determined cultivability after cell sorting. From these analyses, the coexistence of three distinct subpopulations was observed: intact/culturable, permeabilized/dead, and potentially injured with decreased culturability [55].

Sunny-Roberts and Knorr monitored changes in membrane integrity using propidium iodide and esterase activity with carboxyfluorescein diacetate of* L. rhamnosus* in response to osmotic stress, which is a condition that this bacterium has to challenge in natural habitats as well as in food formulations and processes where a probiotic bacterium is used. By FCM and conventional culture techniques, it was found that this microorganism is able to tolerate even extreme sucrose concentrations [83], and such a characteristic is exploitable for its use in food processing and formulations.

In another study, FCM was employed to assess physiological responses of the industrially important bacterium* B. licheniformis*, used in bioremediation processes. Using the same technique, researchers have monitored population dynamics of continuous cultivations, responses to starvation conditions, and responses to glucose and lactose pulses [84, 85].

The heterologous expression of proteins in* E. coli* is a routine method for obtaining large amounts of recombinant proteins. As this process may represent a stressful condition to* E. coli* cells, Borth et al. analyzed by FCM the physiological changes in response to the metabolic stress that represents the production of foreign proteins at high yield. They measured total DNA and RNA content (indicators of cell division and protein synthesis activity, resp.), total protein content (cell size), and intracellular SOD content (foreign protein). They found that after induction of foreign protein synthesis, cells increased their biomass but did not divide. Thus, they conclude that it is important to fine-tune the expression system in order to prolong lifetime and therefore production yield [86]. Thus, FCM could help monitoring physiological changes in response to fine-tuning. Trip et al. developed an elegant, FCM-FACS based system to detect and separate* B. subtilis* cells that secrete heterologous proteins in large amounts. This system is proposed to be used in screening for biotechnologically important secreted proteins from genomic libraries of uncharacterized bacteria. Such a system consists of a* phtrA-gfp* reporter system, whose expression is activated by the CssRS secretion stress response; thus, when the reporter* B. subtilis* strain over produces and secretes proteins, the CssRS system is activated and promotes the expression of* gfp* under control of* htrA promoter*, thus producing a green florescent* B. subtilis* cell that can be separated by FACS for further propagation and analysis [87].

FCM has been also employed to characterize the physiological response of bacteria to organic compounds, such as phenol and toluene. A study that aimed to investigate these points was carried out by Wiacek et al. [33]. It was observed that a culture of* Cupriavidus necator* exhibited a heterogeneous response to harmful phenol concentration treatment determined by measuring chromosomal DNA and PHB content (with DAPI and Nile red, resp.), and after sorting different responding subpopulations they performed functional proteomics analysis [33]. The same group also investigated later the mechanism by which some bacteria uptake toluene. By means of a fluorescent labeled toluene analogue dye (NBDT), they measured toluene uptake using FCM, and after cell sorting, proteomics analysis revealed the presence of toluene transporting porins in* P. putida mt-2* when grown on toluene but not when grown in glucose [88].

The latest works exploited the advantage that cell sorting offers, which includes further analysis of subpopulations, either for obtaining more accurate results than those obtained by analyzing batch cultures or for comparing and characterizing the physiological state of different subpopulations.

### 2.4. Host-Pathogen Interaction

FCM is an automated technique suitable for studying bacterial interactions with host cells, useful for evaluating exclusively the interaction process or even for characterizing such interaction.

Analysis of expression of GFP reporter fusions during analysis of host-pathogen interactions is one of the most common methods employed; following this approach neither the physiology nor the pathogenesis course is altered [89–93].

Stapleton et al. established and standardized the use of FCM to analyze the relative adherence of uropathogenic* E. coli* to clinical samples of exfoliated vaginal epithelial cells (VEC). The researchers employed three GFP-expressing* E. coli* strains isolated from women with urinary tract infections, expressing different types of fimbria and with different* papG* classes. They counted PI stained VEC's, measured adhered bacterial associated GFP fluorescence, and determined the percentage of positive VEC's and the number of adhered bacterial cells. Their results showed different adherence patterns among* E. coli* strains expressing different adhesins [94].

Hara-Kaonga and Pistole's study is another example of the application of FCM to detect bacteria-host cell interactions. They were interested in clarifying whether* ompC* and* ompD* from* Salmonella enterica* were involved in the recognition by macrophages and epithelial cells. Specifically, the researchers analyzed whether these cells differentially recognize single and double* ompC ompD* null mutants. To this end, bacterial cells were stained with FITC and were later incubated with macrophages or epithelial cells. Then, the fluorescent human cells were detected by FCM and the number of adhered bacterial cells was determined. Their results showed that OmpD, but not OmpC porin, is involved in the recognition of* S. enterica* serovar* typhimurium* by human macrophages and epithelial cells [95]. The same authors later reported a method for assessing the adherence of* S. enterica* to host cells by measuring FITC associated fluorescence of bacteria and lipophilic dye PKH-26 associated fluorescence of eukaryotic cells, therefore eliminating the inaccuracies due to bacterial cell aggregates that mimic forward scattering of eukaryotic cells [96].

Another application of FCM in the study of pathogen-host interaction is analyzing genes that are specifically expressed when this interaction takes place. An example was reported by Bent et al. who investigated the expression of* yspP* and* orf6* genes, codifying, respectively, an effector protein secreted* by* and a structural component* of* the T3SS (type 3 secretion system) apparatus. The* yspP* and* orf6* expressions were determined by GFP reporter fusions to those gene's promoters. The reported* Yersinia enterocolitica* cells were used to infect mice. Infected murine cells were subjected to flow cytometric analysis in order to determine GFP fluorescence associated to* yspP* and* orf6* genes expression; this approach combined with qRT-PCR demonstrated that Ysa T3SS is expressed in infected mice [97].

With the purpose of separating cells of interest for further analysis using FACS technology, reports have been published that analyze gene expression during host-pathogen interaction. One common approach was to make a fusion library of chromosome fragments and to clone the fragments in a vector carrying a promoterless* gfp* gene to capture promoters. The approach is the following: bacterial cells transformed with plasmids that express GFP when interacting with host cells are separated by FACS and are later recovered, and their promoter is then at least partially sequenced to identify genes expressed during host-pathogen interaction. With this experimental approach, Barker et al. investigated the genes of* Mycobacterium marinum* that are differentially expressed during macrophage phagocytosis. They sorted fluorescent phagosomes, and after 2 to 3 days after infection, they separated vesicles containing single cells; following this approach, they identified 12 clones containing GFP fusions differentially expressed including membrane proteins and biosynthetic enzymes [98]. Following a similar approach, Wilson et al. analyzed the* in vivo* transcriptional response when* Listeria monocytogenes* infected a murine macrophage model [99].

Thus, flow cytometry offers a rapid, accurate, and reproducible method to quantify the number of wild type or mutant bacterial cells adhered or phagocyted by host cells, with technical complications that each bacterium and host cell type represents. On the other hand, the expression of selected genes during host-pathogen interaction can be assessed, or even genes participating in infection processes can be identified using FCM coupled to cell sorting for further analysis. Although fluorescence microscopy could be an alternative for these purposes, FCM offers the advantage of analyzing and counting a high number of cells, thereby improving the statistics. However, it must be pointed that FCM does not provide information regarding the distribution or location of bacteria within the host cell, and hence a microscopic analysis could be an excellent complement to this technique.

The pathogenic bacteria* Shigella flexneri* are capable of causing dysentery by invading the epithelial cells of the colon. To identify the factors that allow this bacterium to infect the epithelial layer, an FACS based approach was employed to sort mutant clones incapable of spreading within and between epithelial cells. At least three different classes of mutants were identified with this approach, namely, those that presented an altered lipopolysaccharide structure, clones that were affected in intracellular motility, or those that exhibited defect in its capacity to invade the cell hosts [100]. With the purpose of understanding the molecular basis of bacterial virulence, genetic approaches combined with FCM have been applied to identify and isolate nonvirulent bacteria. In a recent study, a liquid culture of* Vibrio cholerae* was subjected to random transposon mutagenesis with the purpose of generating mutants deficient in virulence activation. The entire population was subjected to FACS to successfully separate the cells that were deficient for virulence activation [101]. In another report, FACS was applied to separate recombinant* Mycobacterium* capable of expressing high levels of a foreign ovalbumin epitope; notably, the sorted clones were more efficient in inducing an immunogenic response. These studies demonstrated that recombinant* Mycobacterium* has the potential to be employed as vehicles to delivering pathogen antigen peptides and inducing systemic and mucosal immune responses [102].

### 2.5. Analysis of Bacterial Physiology at a Single-Cell Level

FCM has also been applied to understand the physiological changes occurring in bacterial cells in response to environment changes. One of the most regulated processes in bacteria is cell division. In Gram-positive bacteria, the set of proteins required for cell division is known; these cell division proteins (CDPs) must act in a specific yet known order. Trip et al. were interested in investigating if transcription of those CDPs is the underlying process that regulates speed of cell division. To investigate this aspect, transcriptional gene fusions between CPD promoters and the GFP encoding gene were recombined into a neutral locus of the chromosome of* B. subtilis*. The GFP-fusion containing strains were employed to measure by FCM and qRT-PCR if CDPs expression varies or not under different culture conditions that modify growth rate. Constant expression levels of the fusions were found when tested independently of the growth rate and cell cycle; therefore, it was concluded that cell division in* B. subtilis* is regulated mainly at a posttranslational level and is influenced by other factors that do fluctuate, such as metabolic state and substrate availability, but not by transcription [103].

DNA staining and subsequent FCM quantitative analysis has been applied to gain insights into mechanisms that underlie cell cycle regulation and chromosome biology in bacterial cells. Such knowledge impacts on the understanding of the life cycle of the bacterial cell and can be used to determine cell states quickly and thus predicts the metabolic and survival behavior of microorganisms [4, 104]. DNA quantification patterns are indicative of the number or chromosomes, and by FCM analysis information can be obtained about the number of individual cells that possess an *n* number of chromosomes in an asynchronous growing population. These patterns are characteristic of distinct bacterial species and change under different environment conditions (reviewed by [105]). When studying protein production, function, and localization in batch cultures, it is important to synchronize the cells. FCM analysis of DNA stained with chromomycin has been used to ensure the quality of synchronous population of* Caulobacter crescentus* for studying morphogenesis and cell cycle regulatory proteins that function along the cell cycle [106]. DNA staining histograms obtained by FCM, after treating the cells with drugs that inhibit replication initiation and cell division, are an approach employed to find the number of chromosomes per cell. Thus, variations in these distribution patterns may be analyzed as a function of different conditions and genetic backgrounds to gain insights into cell cycle regulation [107, 108].

There is also a wide interest in studying bacterial differentiation due to the impact of resistant spores and antibiotics resistance related to competence development over clinical, sanitary, and biotechnological issues.* B. subtilis* is a suitable model for studying the molecular mechanisms underlying natural competence development and sporulation [109]. Both developmental pathways are interconnected and appear to be mutually exclusive. Chung et al. (1994) published one of the pioneering works that applied FCM to investigate the heterogeneous development of spore differentiation pathways in* B. subtilis*. In this work, using* lacZ* reporter fusions with early spore development genes* spoVG*,* spoIIG*, and* spoIID*, they measured *β*-galactosidase activity at the single-cell level, revealed by the hydrolysis of the fluorogenic compound C8-FDG (5-octanoylaminofluorescein-di-*β*-D-galactopyranoside) that releases C8-fluorescein which emits green light when excited at 488 nm. They found with this approach that, within a culture of sporulating* B. subtilis* cells, there are two distinct subpopulations and only one of them initiates and concludes with the spore developmental program [110]. FCM and fluorescent reporter fusions to promoters have been employed to gain insights into the pathways that control sporulation and competence development and in the molecular mechanisms by which these processes occur in heterogeneous populations within an isogenic culture; this is important because only a small fraction of cells may experience those developmental processes. Smits et al., employing a (ComK activated) *P*_com*G*_-*gfp* to analyze competence development at single-cell level by FCM, showed the importance of the transcriptional autostimulation of com*K* in the development of competence and also demonstrated that this state occurs in a population of cells that reach a threshold level of ComK [111]. Later, following a single-cell analysis approach, that analyzed the expression of a *P*_spo*IIA*_ *-gfp* fusion activated by Spo0A, allowed them to conclude that the autostimulatory activation of Spo0A is responsible for the bistable expression pattern in sporulating cultures [112]. Using the same reporter fusion, this research group performed single-cell analyses on the expression patterns of both competence development and sporulation of a* B. subtilis* culture of a 1 : 1 mix spore induction: competence induction in chemically defined liquid media and also in biofilms. They found that, in the 1 : 1 mix, both processes are sequentially initiated; first competence develops and sporulation activates later. In another experimental approach, it was found that a small fraction of spores were also capable of acquiring competence. They also found that sporulation is more effectively initiated in biofilms than in planktonic cells. Moreover, this group reported that, under conditions that do not usually trigger sporulation or competence, there are few cells that form spores or that become competent respectively; that is, both differentiation pathways are noisy. Of note, these analyses were only possible by employing FCM* noise measurement*, since at least 100 000 cells must be analyzed under rigorous gating to detect positive cells [109]. Later, this group identified RapH as a novel factor involved in the temporal separation of competence and sporulation. They observed, by using approaches such as single-cell analysis, that the overproduction of RapH provokes a drastic reduction of competence and sporulation gene expression and that* rapH* genetic disruption causes a significant increase in the frequency of cells simultaneously expressing both sporulation and competence fusion reporters; therefore, in a* rapH* mutant sporulation initiation and competence development are no strictly separated [113]. The mechanisms governing the production of exoproteases in nonsporulating cells of* B. subtilis*, within heterogeneous populations, were analyzed by FCM using strains harboring GFP reporter fusions. It was found that only a fraction of the vegetative cells turned on the expression of the bacillopeptidase (*bpr*) and subtilisin (*aprE*) encoding genes under control of the regulator DegU [114].

In a different bacterium, Stecchini et al. [8] investigated the effects of changes in humidity and viscosity in growth cultures of* B. cereus*, a human pathogen [77] over cell motility, spore dimension, and thermal resistance. Such parameters were measured by forward and side scattering (assess dimension) and propidium iodide (thermal damage) [8].

Cronin and Wilkinson, using differential staining, microscopy, and FCM analyses, reported the establishment of a new methodology to identify, quantify, and assess changes in permeability and metabolism of germinating* B. cereus* endospores. This methodology consisted in FCM single-cell level measurement of CFDA/Hoechst 33342 to estimate overall germination rate; and by measuring side-scatter and SYTO9 staining, they quantified ungerminated, germinating, and outgrowing endospores [115].

The list of discoveries enriched and supported by cytometric analysis related to physiological bacterial functions is still growing. Another interesting issue is* quorum sensing* (QS), a way by which bacteria communicate by secreting and responding to signal molecules or autoinducers. Such processes are relevant because in pathogenic bacteria certain virulence factors encoding genes are regulated by* quorum sensing* [116]. Using FCM analysis of reporter strains expressing promoter-*gfp* fusions, Anetzberger et al. monitored the induction/repression of autoinducer-regulated genes in the shrimp pathogen* Vibrio harveyi*. It was found that, besides luminescence, exoprotease gene expression is also regulated by cell density and evidenced by single-cell analysis of simultaneously assessed luminescence and exoprotease expression which they observed functional heterogeneity within the population [117].

In nature, most bacteria exist as aggregates surrounded by an extracellular matrix that is in a biofilm [118]. Biofilms are conformed by specialized subpopulations, with different physiological characteristics compared to those exhibited as planktonic cells [25, 26, 119]. In several contexts, including clinical, industrial, and environmental ones, there is a great interest in understanding how a biofilm is formed and how the cells composing it behave [119].

Given the nature of the biofilm, microscopy has been used as the choice method to analyze the structure, localization, or distribution of the biofilm components; however, FCM has also been successfully utilized for analyzing the physiological conditions of the cells in a biofilm [25]. A requirement for FCM analysis is that cells must be free, in a suspension; to accomplish this, the biofilm has to be disrupted, for instance by passing it by trough a pipette or a needle or by mild sonication [25, 120].

The importance of heterogeneous subpopulations in biofilm formation, using* B. subtilis* as model, has been investigated by FCM. Using this technique, it was discovered that the specialized matrix which is essential for biofilm formation is composed by TasA amyloid-like fibers, exopolysaccharide, and hydrophobin BslA and is produced by a specific subpopulation [27, 121–125]. Garcia-Betancur et al. reported the use of fluorescence microscopy combined with FCM to visualize and quantify the subpopulations of matrix producers and surfactin secretors (signaling molecule that triggers differentiation of matrix producers) within biofilms of* B. subtilis*, with the use of fluorescent reporter fusions with promoters of genes required for matrix (*P*_*tapA*_-CFP) and surfactin (*P*_*srfAA*_-YFP) production. They found that the reporter fusions are expressed only in certain subpopulations, under biofilm formation induction, and in the case of the double-labeled strain showed a single population of fluorescent cells expressing both CFP and YFP, which indicates that in the same population both differentiation pathways are coordinately activated [25].

Using the same microorganism, Marlow et al. reported another type of specialized cells involved in biofilm formation. These authors applied FCM and microscopy to detect exoprotease-producing cells within the biofilm. To this end, fluorescent proteins fusions to *P*_*bpr*_ as reporter of expression of exoprotease-encoding gene* bpr* and other fluorescent reporter fusion *P*_*tapA*_ *-mKate2* to monitor matrix producing cells were employed. It was found that the number of exoprotease producing cells increased as the biofilm matures and that exoprotease production is dependent on the levels of the response regulator phosphorylated DegU (that controls swarming motility, biofilm formation, and exoprotease production); moreover, it was found that exoprotease producing cells arise from both matrix-producing and nonproducing cells. Using microscopic analysis of a cross-section of a mature biofilm, they found that the subpopulation that produced exoprotease is enriched in the air interface more than that in the agar interface [126].

Although the above-mentioned works were performed* in vitro*, there are also* in vivo* studies; for example, Beauregard et al. demonstrated that* B. subtilis* colonizes* Arabidopsis thaliana* roots forming biofilms. This colonization has beneficial consequences for plant development. Although the usefulness of this bacterium as a biofertilizer is well known, Beauregard's results established the importance of biofilm formation in plant colonizing. They determined by FCM that plant polysaccharides, like arabinogalactan, pectin, and xylan, play signaling roles during biofilm formation and as a source of sugars for the synthesis of extracellular matrix. This was made by measuring the levels of fluorescence of biofilm forming cells expressing the *P*_*tapA*_ *-yfp* fusion [127]. These results were consistent with* in vitro* pellicles formation assays and* in vivo* biofilm formation as was observed by fluorescence microscopy.

*B. licheniformis* is a useful bacterium for treating waste residues in water generated from alimentary industrial processes and is therefore important to optimize culture conditions for improved cell aggregation and biomass separation. da Silva et al., using FCM combined with biomass quantitation and confocal microscopy, established such optimal conditions, by assessing changes in the limiting nutrient, dilution rate, and agitation intensity looking for those that gave the cell aggregates where the majority of cells were metabolically active [128].

The employment of FCM in studying biofilms is very useful but not sufficient. Although this technique contributes to the statistics and accuracy of determinations, at single-cell level, it does not provide information regarding the structure in the biofilm and distribution of the distinct subpopulations.

## 3. Concluding Remarks

FCM is a tool that is currently applied to bacterial analysis from detecting and counting bacteria, to determining changes in cellular functions and metabolic activity, and even in identifying genes that are expressed specifically under certain conditions. Although these analyses can be applied to a sample of cultured bacteria following the classic dilution and plating based methodology, FCM offers real measurements for each cell assessed, and in less time. With an appropriate combination of dyes, the damage caused by antibiotics and other threatening agents may be deduced and the proportion of affected cells determined; moreover, using fluorescent postenzymatic cleavage substrate analogs, metabolic activity can also be determined. FCM is an automated technique: it is time-saving, accurate, sensitive, and as thousands of cells can be processed per second, statistics are improved; this point is relevant and it is what makes FCM indispensable for certain purposes, such as identifying and quantifying cells present in very low abundance in a population. However, perhaps the most remarkable potential of FCM when coupled to cell sorting is that offers a unique opportunity to separate specific subpopulations of bacteria that exhibit differential physiological states, from entire cultures. Applying proteomics and/or transcriptomic analysis to characterize such subpopulations makes this approach a powerful tool to understand the cellular and molecular mechanisms that govern cell differentiation in bacteria. Finally, it must be pointed out that FCM and FACS require expensive equipment and skilled personal to operate it and interpret the results. With the appearance of low cost equipment in the market, it is expected that, in the near future, more studies can be carried out to study physiological responses in bacteria using these powerful approaches.
